# Differential Measurement of Involuntary Breathing Movements

**DOI:** 10.3390/bios15020087

**Published:** 2025-02-05

**Authors:** Jacob Seman, Carlos Rodriguez Amaro, Lillian Tucker, Jordan M. Fleury, Keegan Erickson, Gannon White, Talles Batista Rattis Santos, Michelle M. Mellenthin

**Affiliations:** 1 Department of Computer Science and Engineering, Colorado Mesa University, Grand Junction, CO 81501, USA; 2 College of Engineering and Applied Science, University of Colorado, Boulder, CO 80309, USA; 3 Engineering & Physics Department, Whitworth University, Spokane, WA 99218, USA; 4 College of Science, Technology, Engineering & Mathematics, Eastern Washington University, Cheney, WA 99004, USA; 5 Department of Kinesiology, Colorado Mesa University, Grand Junction, CO 81501, USA; 6 School of Health & Human Performance, Northern Michigan University, Marquette, MI 49855, USA

**Keywords:** accelerometer, breathing movements, differential measurements

## Abstract

Free divers are known to experience a physiological response during extreme breath holding, causing involuntary breathing movements (IBMs). To investigate these movements, a low-cost multi-core ESP32-Pico microcontroller prototype was developed to measure IBMs during a static breath hold. This novel device, called the bioSense, uses a differential measurement between two accelerometers placed on the sternum and the xiphoid process to acquire breathing-related movements. Sensor placement allowed for data acquisition that was posture- and body-shape-agnostic. Sensor placement was also designed to be as non-intrusive as possible and precisely capture breathing movements at configurable sampling rates. Measurements from the device were sent over WiFi to be accessed on a password-protected webserver and backed up to a micro-secure digital (microSD) card. This device was used in a pilot study, where it captured the various phases of breathing experienced by recreational free divers alongside a force plate measurement system for comparison.

## 1. Introduction

The number and types of devices for tracking human movement have greatly increased in recent years. In these devices, one of the most commonly used sensors is accelerometers. For example, accelerometers have been used in combination with other sensors to help classify the kind of activity human subjects were performing [1]. Other studies have used accelerometers on smartphones to recognize various activities of daily life [2,3]. In many cases, there are purpose-built devices that utilize accelerometer information. For example, Kamio et al. [4] presented the design of a device which was used to track the movement of a human subject’s arm using accelerometer data.

Accelerometers have also been popular sensors to take measurements relating to respiration and posture. Respiration rate is a popular health metric which can be measured with accelerometers [5,6,7,8]. Based on accelerometer placement, such as those placed on the wrist, additional sensor information may be needed to determine respiratory rate [9]. Santos et al. proposed a method using inertial sensors to track human posture during movement [10].

**Figure 1:**
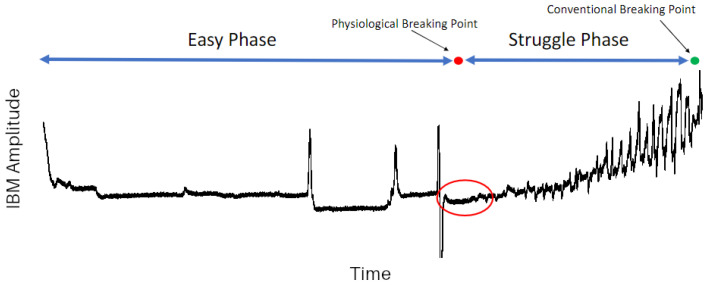
Typical IBM amplitude plot with physiological breaking point indicated (red).

In this study, a purpose-built device was created with the purpose of measuring physiological information relating to the breathing movements of free divers. During prolonged breath holding, there is a growing urge to breathe accompanied by involuntary contractions of the diaphragm and intercostal muscles [11,12]. These contractions are termed involuntary breathing movements (IBMs). When observing and measuring IBMs, there are four main points: the easy phase, the physiological breaking point, the struggle phase, and the conventional breaking point. The easy phase is the duration of breath holding before the first IBM occurs. The physiological breaking point is the point in time where the first IBM occurs. The struggle phase is the duration of time after the first IBM occurs. The conventional breaking point is the point in time where the breath hold ends and breathing is reinstated. Figure 1 shows a representative sample of IBM force over time measured by force plates during a maximal breath hold.

In most devices that are used to measure IBMs, belt transducers track the expansion of the chest [13,14,15]. However, the use of a belt tends to be size-restrictive and very sensitive to auxiliary movements beyond postural adjustments. In lieu of a respiratory belt, Fleury et al. [16] and Singh et al. [17] used an innovative approach and had participants lay supine on force plates to investigate IBMs, as shown Figure 1. However, the force plate measurements were prone to signal noise due to postural adjustments throughout prolonged breath holding. In the case of force plate measurements, the resulting data can contain signal spikes not related to IBMs and can lead to an overall drift in the average force as participants adjust their position on the force plates. The force plate approach also has the limitation of requiring subjects to remain stationary.

The purpose of this pilot study was to create a low-cost device (the bioSense) capable of capturing the involuntary breathing movements of a human subject performing an extended breath hold, which is common in free divers. Efforts were made to ensure that the device would minimize artifacts from postural adjustments. The design presented here is readily capable of acquiring data of comparable quality to the force plate technique.

## 2. Materials and Methods

The bioSense device was tested as part of a pilot study on the Colorado Mesa University campus (institutional review board approval number 22–35) on 1 subject as a proof of concept. Features of the bioSense device are detailed in the subsequent subsections, which cover the hardware (Section 2.1), software (Section 2.2), placement and use (Section 2.3), testing (Section 2.4), and signal analysis (Section 2.5).

### 2.1. Hardware Overview

**Figure 2:**
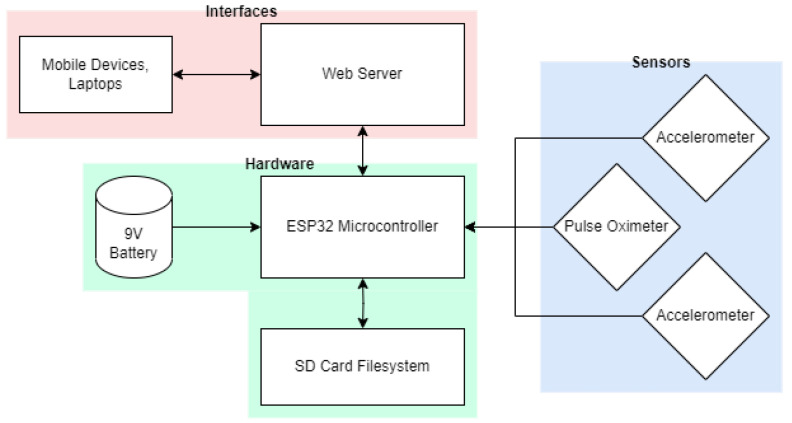
Hardware overview of the bioSense device for involuntary breathing movement measurement is shown depicting the hardware placed on the printed circuit board (cyan), peripheral sensors placed on the subject’s body (blue), and remote interfaces (pink).

The design of the bioSense measurement system is presented in Figure 2. The system incorporates an ESP32 PICO Kit development board (Espressif Systems, Shanghai, China) to collect differential single-axis position data from two I2C accelerometers (MPU 6050, Adafruit, New York, NY, USA) at a configurable rate of up to 100 Hz. Auxiliary data from a pulse oximeter and heart rate sensor (SEN-15129 which combines the MAX30101 and MAX32664, SparkFun, Boulder, CO, USA) are also acquired at a rate of 1 Hz. All measurements are stored and the data can be accessed from a micro-secure digital (microSD) card (MicroSD TF Card Adapter Reader Module, HiLetgo, Shenzhen, China). The bioSense also has a password-protected web server interface to enable remote starting and stopping during data acquisition. The remote starting and stopping feature is valuable as it keeps the person performing the data acquisition from crowding around the human subject performing the breath hold. If desired, data can also be downloaded securely over WiFi. The device can be powered by a 9V lithium battery for up to 3.5 h.

#### 2.1.1. PCB Design

**Figure 3:**
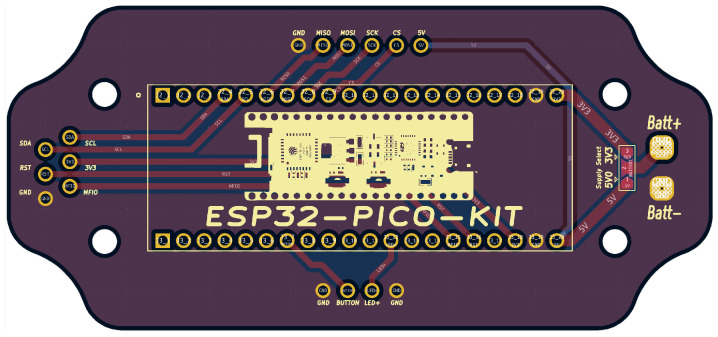
Printed circuit board design layout.

A dual-layer printed circuit board (PCB) was designed in KiCAD (version 7.0.0) to mount the microcontroller into a plastic clamshell enclosure. This PCB design, shown in Figure 3, routes the I2C pins of the ESP 32 to a wire harness for the accelerometers and SpO_2_ meter. It also includes connections for a micoSD card reader, a debugging button, a status LED, and perforated pads for battery power with a solder-bridge option to select 3.3 V or higher voltage supplies. The design shape fits the device enclosure and mounting pattern.

#### 2.1.2. Device Specifications and Bill of Materials

An overview of the bioSense device’s key features and operating specifications is provided in Table 1.

The bioSense is a low-cost device, costing approximately USD 105.02 to create. The bill of materials is specified in Table 2. Some of the items are readily available to be initialized in the code, such as the Adafruit SSD1306 OLED display, and provide an alternative way to interact with the device if the webserver is not needed.

### 2.2. Software Overview

The open-source software of the bioSense device can be readily configured. The tool was developed using Arduino IDE (version 2.0.3) and key features of the software are detailed here.

#### 2.2.1. Dual-Core Task Organization and Timing

The program utilizes Free-RTOS tasks to make use of the two available cores on the ESP32. Free-RTOS is a real-time operating system kernel provided as standard with Arduino libraries. Using task pinning, functions can be assigned to a specific core and will run indefinitely on that core until the task is canceled, similar to the typical program loop, which by default only runs on core #1. This multicore “threading” allows for the grouping of functions to optimize the speed of data acquisition and handling. For example, all custom bioSense functions were written such that all I2C bus devices are handled by core #0, while SPI and analog pin states are handled by core #1. For example, the function “sensorRead” is pinned to core #0, while the function “sdWrite” is pinned to core #1.

**Figure 4:**
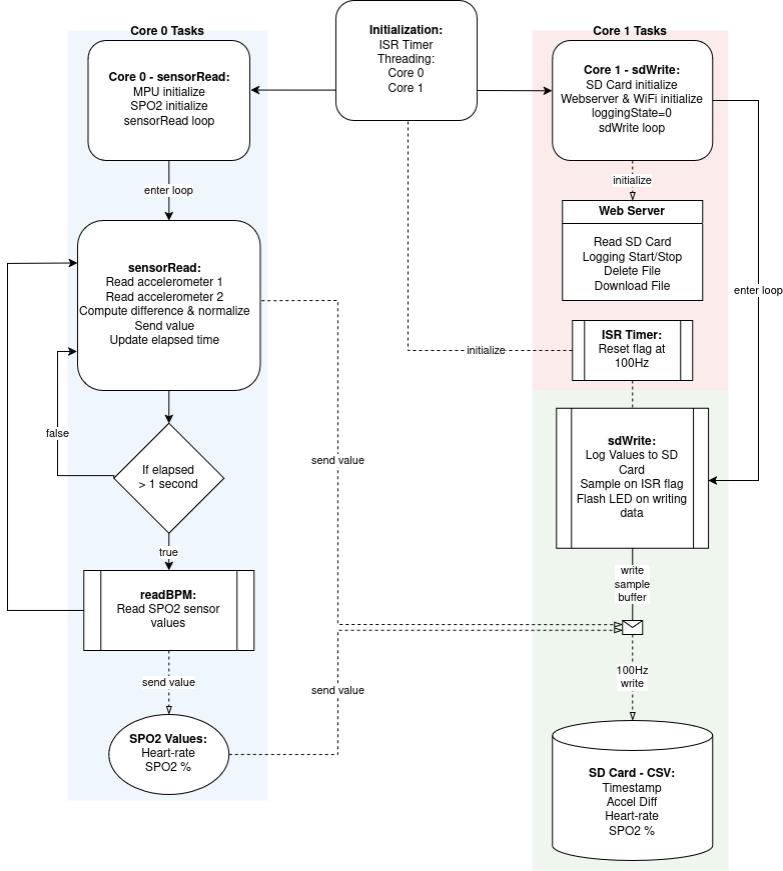
Block diagram illustrating the division of tasks performed by each of the ESP32 cores, with functions grouped by color to denote data storage (cyan), peripheral sensors (blue), and remote interfaces (pink).

Some conflicts can occur with multi-core threading, so care was taken to ensure that no data are written to the same location by both cores simultaneously. In this case, core #0 only reads accelerometer data, and writes temporary data to the “fauxList” class where the most recent 10 accelerometer difference samples are stored. Core #1 reads and averages these data before writing them to the SD card. Because the accelerometer and pulse oximeter readings are performed over I2C, and the SD card writes are performed over SPI, there is no conflict between the two processes. The block diagram in Figure 4 illustrates the program code’s task and function organization.

A hardware interrupt timer running from RAM was used to control event timing and data rate limiting. The ESP32 provides an onboard 4MHz hardware clock that may be used with a dividing value to achieve a specific frequency of operation. Each increment in the hardware timer triggers an interrupt which allows very-low-level increments in data rate counters and the setting of state flags to occur. In this way, the timing and data rate can be precisely defined and controlled at a low level. The interrupt counter is also used in the data output to provide accurate timestamps alongside measurements.

#### 2.2.2. Peripheral Device Management

The bioSense manages four wired peripheral devices: two accelerometers, a pulse oximeter, and the microSD card reader.

To increase the sampling rate to approximately 100 Hz, the associated accelerometer library found in the Arduino IDE should be avoided. Instead, it is better to configure the accelerometers using I2C libraries to enable reading the data directly from the MPU-6050 registers. This process takes 1153 microseconds compared to 5333 microseconds using the standard library. This selective method of reading data allows for measurement data to be read only from the Y-axis register of either accelerometer. Limiting the data to a single axis also helped in rejecting off-axis movement. As an I2C device, these measurements are controlled by core #0.

The pulse oximeter peripheral is used to provide auxiliary information on heart rate and blood oxygenation. The Sparkfun SEN-15129 combines two integrated circuits from Maxim Integrated, the MAX32664 Biometric Sensor Hub, and the MAX30101 Pulse Oximetry and Heart Rate Module. The SparkFun Bio Sensor Hub library is recommended for data acquisition. As an I2C device, these measurements are controlled by core #0.

The microSD card reader provides a filesystem to the microcontroller where data are stored. As an SPI device, it is controlled by core #1. The microSD card in the reader is mounted upon device initialization and remains mounted while powered. At the start of each data acquisition, a comma-separated value file is created and data are written line-by-line to this file at a rate of 100 Hz. After the signal is received to stop logging, the file is closed and a filename counter is incremented for the next log. The microSD card must remain mounted to provide filesystem access to the web server features.

#### 2.2.3. Configuring the Web Server

The web server provides a user interface for wireless interaction with the microcontroller and its features. As soon as the ESP32 is powered on and all other hardware successfully initializes, a WiFi access point is created using the “ESP32WebServer” library. The web server is implemented using a cascading style sheet and basic HTTP functionality.

### 2.3. Human—Device Interface

**Figure 5:**
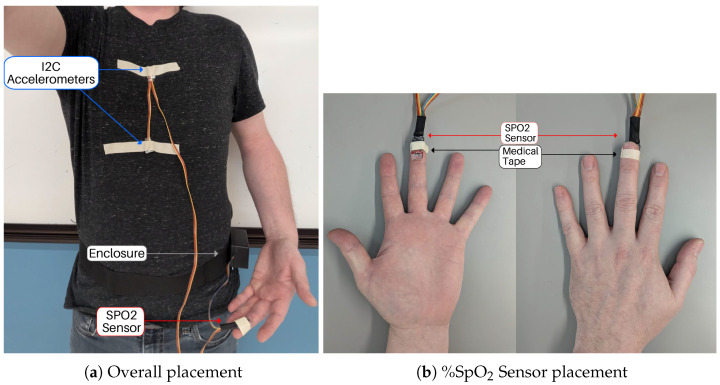
The bioSense device can be placed on the body as shown in (**a**) with the accelerometers placed approximately on the xiphoid process and tip of the sternum. The belt containing the ESP 32 and battery should be worn in a comfortable position, such as around the hip. The %SpO_2_ sensor should be placed on the index or middle finger as shown in (**b**) and can be secured with medical tape.

The differential accelerometer measurement is acquired by taking the difference between two accelerometers placed directly along the midsagittal plane of the subject. The difference between these measurements is averaged over 10 samples and stored to the microSD card filesystem. Accelerometer placement was empirically determined by the research team after observing an extended breath hold and identifying anatomical locations with the most movement. To ensure that sensor placement could be consistent between subjects, distinct anatomical features were selected as the sensor placement points. The xiphoid process was selected as the measurement location, and the sternum as the reference. The placement of the device on a healthy human volunteer is shown in Figure 5.

The accelerometers may be taped down over clothing provided the clothing is not excessively loose-fitting. The SPO_2_ sensor may be placed on the pad of the subject’s index finger using tape or secured using a Velcro strap. To avoid intermittent readings from the SPO_2_ sensor, it is important to ensure the sensor is not pressing firmly into the fingertip pad—just enough pressure to keep the sensor in place without squeezing the fingertip is best. The wire harness is sufficiently long to accommodate any body shape and size and may be oriented so that the subject is most comfortable.

If the subject is laying down, a small enclosure that holds the microcontroller, microSD card, and a 9 V lithium battery power source can be placed next to them. This enclosure is secured to a belt with Velcro so the enclosure can easily be opened and closed or worn around the hips if desired.

#### Web Server Interface Operating Instructions

If using the bioSense with the default settings, persons using the device to record data can navigate to the “Logging” web server tab. By pressing the “Logging Start” button, data will immediately start logging onto the microSD card. The red LED will blink to show the active logging to the microSD card. To stop acquiring data, the user should press the “Logging Stop” button. Data will immediately stop logging and the file will be closed. The red LED will cease blinking to show the device has stopped logging onto the microSD card. Multiple logging sessions can be initiated and closed without overwriting any previous files logged onto the microSD card. It is also possible to download or delete files logged on the microSD card from the web server.

### 2.4. Testing

Aside from the iterative testing performed as part of the bioSense development process, two important tests were conducted. A hardware test was used to determine the current consumption and approximate battery life. Current measurements were acquired using a Keithley DMM6500 multimeter (Tektronix, Cleveland, OH, USA).

Pilot data were collected on two healthy adult volunteers with recreational free-diving experience. The volunteers were asked to lie in the supine position on top of a force plate so that force plate data and bioSense device data could be acquired simultaneously. As a backup to the bioSense measurements of heart rate and blood oxygen saturation, a commercially available fingertip pulse oximeter (FaceLake FL400, Buffalo Grove, IL, USA) was used. Heart rate and %SpO_2_ were manually recorded at 10 s intervals from the backup device. The motivation for capturing these other health metrics was to investigate whether changes in heart rate and %SpO_2_ were associated with the physiological break point of IBM movements.

### 2.5. Signal Processing

The raw differential accelerometer measurements (y) were processed using a sequence of steps to remove movement artifacts and noise unrelated to the IBM signal. First, the measurements were truncated to remove the sitting motion of the human subject and start at the beginning of the breath hold. Then, a zero-phase shift, 12th-order lowpass Butterworth filter (50 Hz) was applied to remove high-frequency noise and then downsampled from approximately 1000 Hz to 100 Hz. Afterwards, the data were normalized using the following formula:

(1) $$\begin{array}{c} y_{fig10}=\frac{y_{raw}-mean\left(y_{easy}\right)}{max(y_{raw}-mean\left(y_{easy}\right))} \end{array}$$

Using the normalized data, the easy phase and struggle phase of the IBM movement can be identified. A fast Fourier transform (FFT) was performed for the total duration of the easy phase and struggle phase to determine the dominant frequencies during each phase. Next, a zero-phase shift, 5th-order bandpass Butterworth filter (0.5 Hz, 3.5 Hz) was applied to remove low- and high-frequency noise. In post-processing, the filtered data were squared to remove negative components:

(2) $$\begin{array}{c} y_{filt}=y_{filt}^{2} \end{array}$$

$y_{filt}^{2}$ was used to find a trendline of the signal’s magnitude, as determined by the *trenddecomp* function in MATLAB software. This function finds trends in a vector of data using singular spectrum analysis, which assumes an additive decomposition of the data such that

(3) $$\begin{array}{c} A=L+S+R \end{array}$$

where L is the long-term data trend, S captures the oscillatory trend(s), and R is the remainder. By applying this technique to measured data of an extended breath hold of a recreational free diver, the overall increase in the magnitude of spectral components can be determined. To detect time-dependent changes in frequency, filtered data were visualized using the *spectogram* function in MATLAB (version 2024b).

## 3. Data and Results

### 3.1. Battery Life Approximation

**Figure 6:**
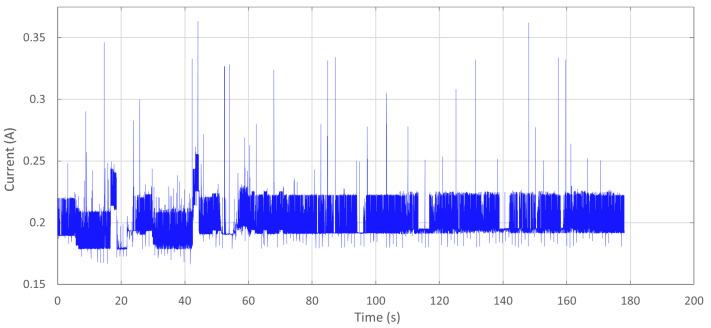
Device current draw while performing each possible functionality action over time.

The device was found to consume an average of 197.5 mA when idle, actively logging, serving data file downloads, and deleting files. The device’s current draw, shown in Figure 6, was measured during these activities to determine when the current draw changed significantly, if at all.

Note that the current draw stays consistent throughout the duration despite various actions being performed by the microcontroller. While some actions did cause instantaneous peaks for starting and stopping events, these peaks failed to impact the average current draw significantly. When using an Energizer I522 9V lithium pile battery discharged at 200 mA to 5.4 V, the battery’s capacity is rated to provide 700 mAH. This capacity at this current will provide 3.5 h of runtime.

### 3.2. Pilot Study Measurements

**Figure 7:**
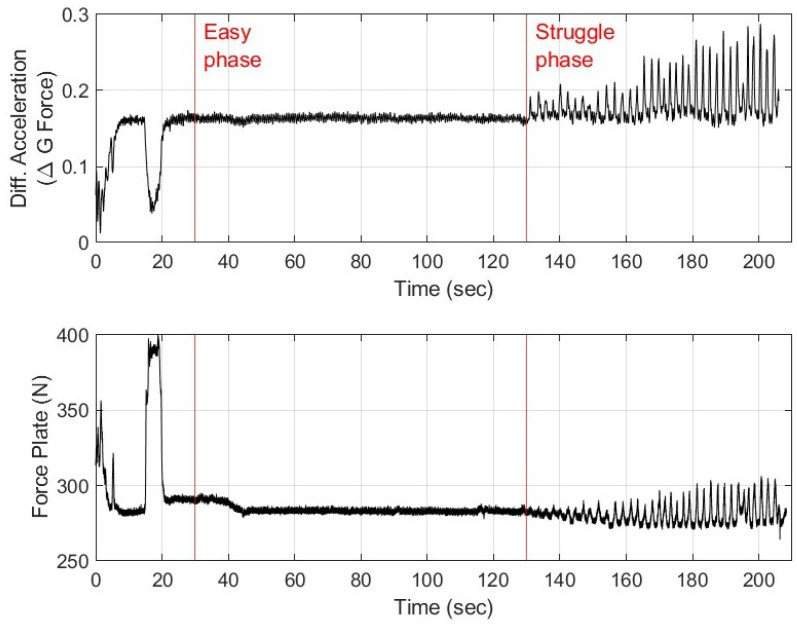
BioSense device differential acceleration measurements as compared to force plate measurements for a healthy human volunteer performing an extended breath hold. Vertical red lines are used to mark the start of the easy phase and struggle phase.

The subject was able to hold their breath for nearly 80 s into the “struggle phase”. The raw data showing differential accelerometer measurement and force plate data for the subject are shown in Figure 7.

A comparison of IBM data simultaneously acquired on a force plate and the bioSense device in Figure 7 reveals that the differential accelerometer data were also able to capture the physiological involuntary breathing movement response. The stages of the extended breath hold are identified at the same time points by both the force plate and the differential acceleration technique. However, the IBMs were detected with greater relative amplitudes from the bioSense as compared to the force plate. Figure 7 marks the onset of the easy phase at 30 s and physiological breaking point or the start of the struggle phase at 130 s. In the first 30 s of data collection, the subject arched his upper back and rocked his shoulders firmly into the force plate as part of a postural adjustment to increase comfort during the extended breath hold. The force plate registered this as a large spike, and the bioSense captured this movement as a decrease from the baseline as the chest was lifted rather than contracted during involuntary breathing movements.

Intermittent %SpO_2_ and heart rate data were collected during the maneuver due to poor sensor positioning. However, the bioSense recorded a heart rate of 116 beats per minute (BPM) at the start of the early phase. The reading at this point in time has good agreement with data recorded from the back-up FaceLake FL400 pulse oximeter, which read 122 BPM. The average heart rate during the struggle phase was 136 BPM as measured by the SEN-15129 sensor on the bioSense and 98 BPM from the FL400. The FL400 showed a clear decrease in %SpO_2_ from 97% at 130 s to 86% at 210 s. Data recorded more than 20 s into the breath hold by the SEN-15129 were inaccurate and indicated poor positioning by bioSense. These erroneous readings may have been caused by the movement of the human subject at the start of the breath hold, repositioning the sensor.

### 3.3. Analysis of Acquired Data

**Figure 8:**
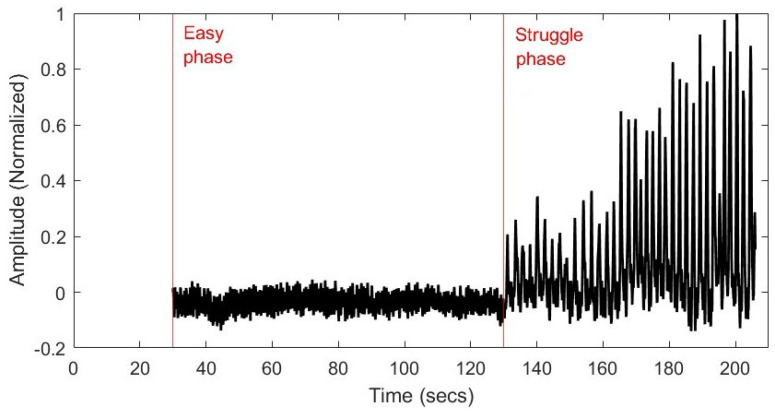
Normalized data from pilot study test subject during an extended breath hold. Data were truncated to remove recorded postural adjustments occurring prior to the start of the breath hold.

As the bioSense was developed as a purpose-built prototype, the differential accelerometer data were analyzed to determine which physiologically relevant features of interest could be detected. Since data from the second human subject did not contain multiple involuntary breathing movements in the struggle phase, only Subject #1’s data were analyzed (Figure 7). The data shown Figure 8 present the normalized data and clearly identify the “easy phase” and “struggle phase” of the extended breath hold.

**Figure 9:**
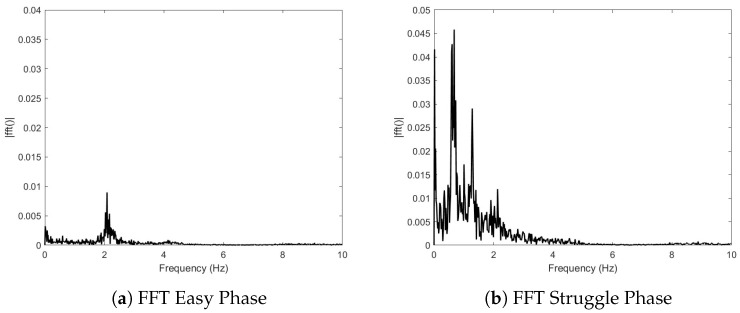
(**a**) The FFT identifies a peak at 2.090 Hz. (**b**) The FFT identifies peaks at 0.677 Hz, 1.285 Hz, and 2.137 Hz.

In Figure 9, the FFT was used on the normalized data to identify the spectral components of the signal. In Figure 9a, the peak is located at a frequency of 2.090 Hz or 125 BPM, which is within 8% of the bioSense SEN-15129 measured heart rate at the start of the “early phase”. This plot also shows very little noise was acquired along with the signal. From Figure 9b, it is seen that the FFT identitifed peaks at 0.677 Hz, 1.285 Hz, and 2.137 (127 BPM). Additionally, 127 BPM is within 7% of the average bioSense SEN-15129 sensor data during this phase.

**Figure 10:**
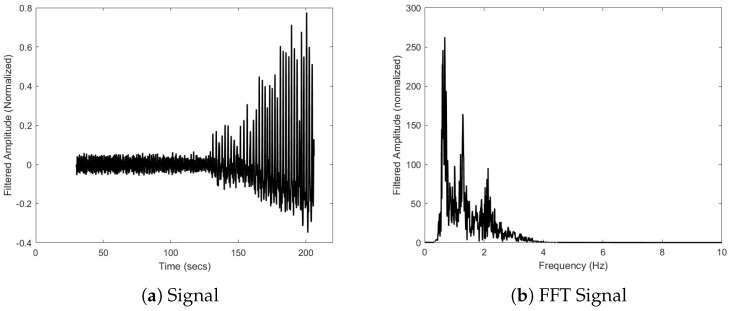
The (**a**) filtered signal and (**b**) FFT after applying a 5th-order digital Butterworth bandpass filter (corners at 0.5 Hz and 3.5 Hz). Frequencies identified in the FFT are 0.676 Hz, 1.284 Hz, and 2.133 Hz.

Figure 10a shows the filtered signal after the Butterworth filter was used to remove signal noise and offsets. Figure 10b confirms the removal of the very-low-frequency noise. The same peaks are identified between Figure 9b and Figure 10b to less than 0.2%.

**Figure 11:**
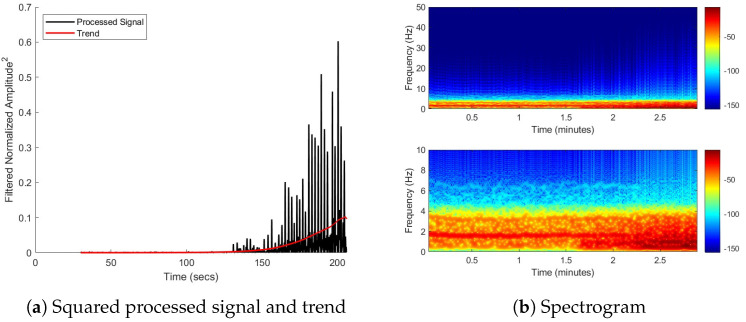
Signal analysis for (**a**) trend in frequency component magnitude and (**b**) time-dependent frequency, or a spectrogram.

Figure 11 uses two different methods to look for changes in the spectral content that could be used to better understand the struggle phase of the IBM. The trendline in Figure 11a indicates that the magnitude of the spectral components increases. The spectrogram in Figure 11b shows an increase in the magnitude of the 0.676 Hz and 1.284 Hz frequencies at the onset of the struggle phase. It is important to note that the time axis of plots in Figure 11b are in reference to the start of the breath hold instead of the start of data acquisition.

## 4. Discussion

The results from this study (Figure 7) show that differential accelerometer measurements from the bioSense device can be used to acquire involuntary breathing movements that are comparable to the force plate technique [16,17]. Specifically in Figure 7, it can be seen that the peaks in the force plate data immediately coincide with the bioSense device readings. In regard to large movements (when the subject was moving prior to the breath hold or 0–20 s), there is an inverse relationship between the data, which can be explained by measurements occurring on the dorsal side (force plate) versus ventral side (differential accelerometer) of the body.

FFT analysis in Figure 9 and Figure 10 shows that with some minor modification, the bioSense could be capable of determining average heart rate without the use of the SEN-15129 sensor. The FFTs also show two frequencies relating to the onset of IBM movements: approximately 0.68 Hz and 1.28 Hz. In addition, Figure 11 shows the ability of the device to capture the increase in the magnitude of the IBM signal and onset of the movements.

Many purpose-built repository monitoring devices rely on measurements from a single accelerometer to capture the movement of the chest. For example, in a device created to measure breathing patterns during sleep, Ryser et al. used a single accelerometer placed on a band around the thorax [18]. The bioSense incorporates two accelerometers since the ability of the subject to resist breathing is of greater interest than the breath. During the struggle phase of an extended breath hold, the subject’s diaphragm contracts trying to force the subject to breathe. Free divers are trained to resist these contractions, so the contractions increase in magnitude as shown in the latter half of the signal in Figure 10a and corresponding trend in Figure 11a. It is suggested that the increasing frequency and magnitude of IBMs throughout the struggle phase, which is a common trend [15,19], is related to increases in cerebral blood flow and oxygen delivery to the brain [11,19].

Sumbul et al. proposed measuring the movement of the diaphragm using a single accelerometer placed near the diaphragm during a study by acquiring tidal breathing and pulmonary function test results [20]. The accelerometer data from this study tracked with the breathing movements. A similar approach was later used by Pryor et al. in a study of diaphragmatic function in an endurance test of the diaphragm muscle [21]. It is unclear in either study if the researchers were able to determine information about the intensity of the diaphragm contraction. Pryor et al. did observe a decrease in acceleration g-forces over time during the test. Future testing of the bioSense could be carried out to determine if the axial contraction of the diaphragm can be sufficiently measured with a single accelerometer or if the differential measurement technique would be beneficial in eliminating other postural movements from the measurements.

With waterproofing, the bioSense device could lead to a potential configuration that would allow IBM data collection while free diving. With greater human subject movement, additional accelerometers may be needed to track movements which are unrelated to breathing. This could assist in interpreting data collected on free divers. Additionally, some minor modifications and additional testing are still needed before the bioSense can be used underwater. For example, after potting the device in resin to provide waterproofing, it is important to ensure that the enclosure and electronics components can withstand underwater pressures of up to 40 feet. In a future study, testing the device on a group of subjects mimicking free-diving motions would be extremely useful.

The pilot study tests were performed during the device development phase, and only one measurement was presented, which limits the device’s performance conclusions. Expanding the number of samples in future studies will enable the device design to be tested for reliability while the human subject is both stationary and moving. In particular, the reliability of the %SpO_2_ sensor will be a priority, as only intermittent readings were acquired during the pilot test. Additional program debugging modes can also be enabled to allow logging of raw data to directly compare single accelerometer performance and further quantify posture-related movement rejection. This comparison will be an advantageous future step before using the device on human subjects while they are diving and swimming, allowing for an opportunity to refine the device to minimally hinder movement in the concluding study of this project.

## 5. Conclusions

The bioSense device achieved its goal to measure IBMs in a low-cost manner and the results are comparable to other methods [16,17]. This open-source design can be modified by other researchers needing an efficient way to measure movement. The proposed placement of the device ensures that artifacts from postural adjustments are minimized and physiologically relevant IBM data are obtained. Simple digital bandpass filters can be used to further remove unwanted movement and noise from measured data. The design presented here is capable of acquiring data of comparable quality to the force plate technique.

## Figures and Tables

**Table 1:** Specifications of the bioSense device.

Aspect	Value
Power Supply	5 V 2 A USB-Micro, 9 V lithium battery
Runtime	3.5 h
Current Draw	100 to 200 mA
microSD Card Format	5 GB, Fat 32
Maximum Sample	Rate approx 800 Hz
Default Sample Rate	100 Hz (configurable)
Data Point Averaging	10 Samples (default, configurable)
Data Output	4 columns (with timestamp)
Data Format	Comma-separated value, .CSV file

**Table 2:** The bill of materials for the bioSense device.

Item	Quantity	Unit Price (USD)	Cost (USD)
Espressif ESP32 PICO Kit	1	$10.00	$10.00
Adafruit MPU6050 Accelerometer/Gyroscope	2	$6.99	$13.98
Adafruit SSD1306 OLED Display	1	$2.40	$2.40
microSD card reader (SPI)	1	$1.39	$1.39
Energizer 9 V lithium battery	1	$14.97	$14.97
Sparkfun SEN-15129	1	$42.95	$42.95
SPST Switch	1	$0.95	$0.95
Red LED	1	$0.04	$0.04
Enclosure	1	$8.29	$8.29
Dual-layer PCB fabrication	1	$8.87	$8.87
Wiring harness (per ft)	4	$0.24	$0.94
Elastic band (per ft)	4	$0.06	$0.24

## Data Availability

The human subject data are available from Dr. Michelle Mellenthin (mmellenthin@coloradomesa.edu) upon reasonable request. The bioSense device, design, and instructions are available as an open-source design on GitHub: https://github.com/Jbsco/bioSense (accessed on 2 February 2025).
